# Colour Confusion: Reviewing Ambiguities in the Identification and Classification of Chromatophore Deficiencies Among Amphibians

**DOI:** 10.1002/ece3.72149

**Published:** 2025-09-12

**Authors:** John Gould

**Affiliations:** ^1^ School of Environmental and Life Sciences University of Newcastle Callaghan New South Wales Australia

**Keywords:** colour aberration, colour mutation, iridophore, melanophore, pigment, xanthophore

## Abstract

The colour of amphibian skin and eyes (retina and iris) is the result of light interacting with cells called chromatophores, primarily xanthophores, iridophores and melanophores, that can be found in various combinations. Thus, identifying the chromatophores involved in colour abnormalities in this group is challenging, particularly if it is solely based on a visual comparison of atypical versus typical individuals, as light may be interacting with several types of chromatophores at once. As most records of colour aberrations will likely continue to be based on visual assessments without the procurement of tissue samples, given that these observations are generally opportunistic, there is a need for consistency in how they are classified and explained so that the potential chromatophores that are affected can be deduced. Yet, this is complex as the outcome of different chromatophore defects may differ between species, especially when there are spatial differences in where the defects occur between tissue regions or combinations of abnormalities, while in the presence of other chromatophore types that remain unaffected. In this review, I explore the ambiguity and confusion that currently exists within the literature with regard to terminology used to define colour aberrations among amphibians and the specific types of chromatophores that are assumed to be the basis of colour abnormalities based upon visual assessments. I subsequently establish standards for the terminology and description of colour abnormalities among amphibians, specifically those related to chromatophore deficiencies: hypo‐melanism, hypo‐xanthism and hypo‐iridism. This is followed by guidelines for collecting data on the physical manifestation of chromatophore deficiencies to prevent future ambiguity or errors.

## Graphical abstract

The colour of amphibian skin and eyes is the result of light interacting with multiple chromatophores (xanthophores, iridophores and melanophores), leading to challenges when trying to identify which of these cells are involved in colour abnormalities. I explore the ambiguity and confusion that currently exists within the literature with regard to terminology used to define colour aberrations in this group and the types of chromatophores that are assumed to be their basis. This work addresses the need for standards in assessing and describing colour abnormalities among amphibians, specifically those related to chromatophore deficiencies: hypo‐melanism, hypo‐xanthism and hypo‐iridism.

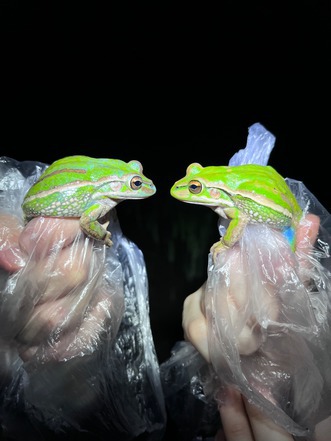

## 1 Introduction

The colouration of externally visible tissues, such as skin, scales, fur, feathers and parts of the eye (e.g., iris), plays a critical role in individual fitness by influencing interactions with conspecifics (e.g., mating selection), heterospecifics (e.g., camouflage) and the environment (e.g., thermoregulation) (Glaw and Vences 1997; Rojas 2017; Sztatecsny et al. 2012; Trullas et al. 2007). The adaptive functions of colour expression are thus under strong selection pressures that should cause the removal of abnormal colouring not typical for a species if it does not provide an advantage or reduces survival and reproduction potential, leading to its rapid loss from the population (Andren and Nilson 1981; Childs Jr. 1953). Given their rarity (Henle and Dubois 2017), colour abnormalities are often not clearly described or considered as critical information, rather curious natural history observations, yet may provide a wealth of information on the health of populations and the pigments/cells involved in colour formation.

The diversity of skin and eye (retina and iris) colours found among amphibians is primarily a manifestation of three main cell types, referred to as chromatophores (Bagnara 1983; Du Shane 1935; Duellman and Trueb 1994). In the skin, these cells are arranged vertically as cell layers in the dermis to form the dermal chromatophore unit (Bagnara 1966, 1983; Bagnara et al. 1968). Melanophores form at the deepest layer of this unit and contain melanin pigment (primarily black or brown eumelanin in amphibians but also yellow‐red pheomelanin) within organelles called melanosomes that give amphibian skin a dark colour (Bagnara et al. 1978; Wolnicka‐Glubisz et al. 2012). Iridophores are located more superficially to the melanophores and contain light scattering/reflecting platelets that give rise to structural colours (Bagnara 1983; Bagnara et al. 1969; Taylor and Bagnara 1972). Xanthophores and related cell types such as erythrophores lie superficially to the iridophores and contain pteridine pigments deposited in organelles called pterinosomes, as well as carotenoid vesicles, which typically give skin a yellow–orange–red colour (Bagnara 1966; Bagnara and Hadley 1973). Additional chromatophores may also be found more superficially within the epidermis layer, such as epidermal melanophores (Bagnara 1983; Bagnara and Ferris 1971). Below the dermis are also deeper skin layers that may contain iridophores, which act as a reflective shield (Nielsen and Dyck 1978). The relative number of each chromatophore type within amphibian skin and their presence may vary between skin regions, individuals and species, including cases where there is one melanophore under each iridophore and one iridophore under each xanthophore or only a single chromatophore type present (Bagnara et al. 1968).

The arrangement of chromatophores within the iris has been far less investigated, but all three chromatophore types can be present and may have a similar layering to that of skin dermis (Barden 1942; Du Shane 1935), but see Barden (1943). Typically, it is the presence of iridophores that gives many amphibians the metallic silver or golden lustre to the iris (Oliphant and Hudon 1993); however, it must be noted that the differentiation between xanthophores and iridophores of the iris is complex (see Oliphant and Hudon 1993). Melanin pigment cells are located within the retina (Sazima 1974), while iridophore‐like cells may also be present in a light reflecting layer at the back of the eye referred to as the retinal tapetum (Zueva et al. 2022).

The colouration of amphibians is thus dictated by the manner in which each chromatophore interacts with the light they receive while in the presence of other chromatophore types, as well as their interactions in terms of the light received by each chromatophore and additive optical effects upon reflection and perception by the viewer, which allows for the production of a diversity of colour expressions. Such interactions between chromatophores make it relatively more difficult but not impossible to visually determine the colour components of amphibian tissues compared with other animals, such as mammals, with only one pigment cell type (melanocyte) containing types of melanin (Slominski et al. 2004). Additionally, many molecules/compounds can influence or contribute to colour expressions beyond chromatophores and their biosynthesised pigments, such as collagen fibres (Nishioka 1977; Nishioka and Ueda 1985a), haemoglobin (Richards and Nace 1983) and dietary carotenoids (Bagnara and Hadley 1973).

Abnormal skin and eye colouration are rare but widely detected among amphibians (Henle and Dubois 2017; Hoffman and Blouin 2000; Vershinin 2004). These result from the reduction, absence or overproduction of one or several chromatophores or their associated organelles and colour‐forming contents (Duellman and Trueb 1994). These defects may be caused by genetic mutations, environmental teratogens such as pollution that can cause pigment degeneration, environmental conditions during development that impact pigment cell migration, or stress/condition and disease (Browder 1972; Henle and Dubois 2017; Kobelt and Linsenmair 1992; Sanabria et al. 2010; Vershinin 2004). Colour abnormalities within wild populations may thus provide an early warning sign of population‐level issues such as inbreeding depression, as well as exposure to environmental stress or contaminants, which can be easily visually detected (Bensch et al. 2000; Vershinin 2004). Thus, it is critical that the chromatic basis of these abnormalities is correctly identified and that different research groups are consistent in their terminology, as this provides continuity in terms of the long‐term monitoring and record‐keeping of target populations for valuable information regarding changes in population health and functioning to be gained. Additionally, colour variants can also provide information on the distribution of chromatophores of target species which are usually masked or difficult to assume based on visual inspections of typically coloured individuals (Turner 2017). This provides unique opportunities to understand the genetics, environmental causes and roles of different colour expressions and can also be useful for investigations in developmental biology (Markert and Ursprung 1971; Nishioka and Hiroaki 1985). Standardised descriptions of chromatophore deficiencies are also needed to reveal important information about the typical distribution of chromatophore cell types across amphibian species, even those that have not been formally assessed, as the colour aberration in one species may be the typical colour of another species.

Yet, it is apparent that there are inconsistencies in the terminology used to describe colour aberrations among amphibians, along with confusion and inaccuracies in the chromatophore deficiencies associated with abnormal colouring when these are based purely on visual assessments of their physical manifestations. Formal histological analyses of skin samples may not be possible given logistical, expertise, time and ethical constraints and may be avoided by some researchers as they require the capture and possible euthanisation of abnormal individuals to collect samples. Thus, there is a need for visual assessments of colour abnormalities to be sufficiently detailed so that the potential chromatophores likely affected can be proposed via comparisons with similar expressions in other species where histological analyses have been performed. Additionally, there is also ambiguity or lack of detail and clarity when it comes to describing abnormally pigmented tissue among amphibians (Baker and Biddle 2020; Brito‐Zapata 2021; García‐Padrón and Bosch 2019). Part of the confusion likely stems from the use of terminology that has been created for animal groups, principally mammals, that possess melanin as the primary pigment type and melanocytes as the primary pigment cell type (Slominski et al. 2004), which has been hastily applied to other vertebrates including amphibians, which possess several pigments and cell types associated with colouration, as noted by Davis (2007). Indeed, the same chromatophore deficiency can have entirely different colour outcomes between tissue regions and species dependent on the presence and combination of other chromatophore types that are unaffected. This is cause for concern as some colour abnormalities may appear to be rarer or more common than they truly are. Additionally, there is a diversity of names to define the same deficiencies, which have either been based on the physical attributes of abnormal individuals or the chromatophores that are likely involved (see Richards and Nace 1983). Standardised terminology and classifications have been established for other animal groups to reduce confusion (e.g., Lucati and López‐Baucells 2017) and there is now a requirement to do the same for amphibians.

The aim of this review is to establish standards for the terminology of colour abnormalities and descriptions of their physical appearance among amphibians, specifically those related to chromatophore deficiencies (hypopigmentation). This extends upon the work of prior reviews (e.g., Richards and Nace 1983), by exploring deficiencies in all three main types of chromatophores, including xanthophores, iridophores and melanophores, across ‘external’ tissues (skin, retina and iris). I review the literature for cases where incorrect and ambiguous terminology or insufficient descriptions have been applied to colour abnormalities among post‐metamorphic individuals and where possible provide corrections or updates. Through this review, detailed explanations of the colours resulting from chromatophore deficiencies, including combinations of deficiencies, have been provided along with guidelines for data collection to reduce future ambiguity or errors. Diagrams (Table 1) have also been provided as a visual guide for understanding the physical manifestation of chromatophore deficiencies in this group. This review is intended to support researchers to more accurately detect and classify chromatophore deficiencies in amphibian populations based purely on a visual assessment of colour abnormalities when tissue analyses cannot be performed. While definitive evidence of the chromatophores involved in colour abnormalities can only be achieved via tissue analysis, visual assessments on their own can still provide valuable information and assist in deducing which chromatophores are likely affected.

**TABLE 1:** Physical manifestations of chromatophore deficiencies among amphibians. Simplified colour expressions of typical frogs (A1, B1, C1, D1) are shown with abnormal colour expressions of these frogs in subsequent rows.

Frog	Colour expression	Skin	Iris	Retina	Notes	Example
Typical Frog A1	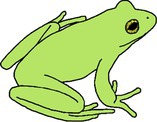	X (✓) I (✓) M (✓)	X (o) I (✓) M (✓)	X (o) I (o) M (✓)	Green skin requires all three chromatophore types Iris gold iridescent	Nishioka and Ueda (1985a)
Abnormal Frog A2	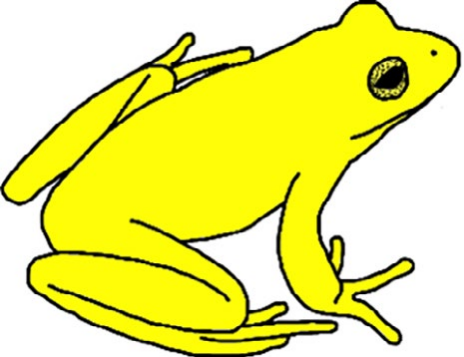	X (✓) I (✓) M (**↓**)	X (o) I (✓) M (✓)	X (o) I (o) M (✓)	Complete hypo‐melanism (cutaneous) ‘Leucism’ Skin is yellow due to xanthophore pigment No iris abnormality	Nishioka and Ueda (1985a)
Abnormal Frog A3	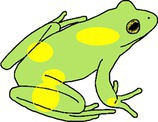	X (✓) I (✓) M (**↓**)	X (o) I (✓) M (✓)	X (o) I (o) M (✓)	Partial hypo‐melanism (cutaneous) ‘Piebaldism’ Patches of normal green skin where melanophore pigment is not affected No iris abnormality	Gould and McHenry (2024)
Abnormal Frog A4	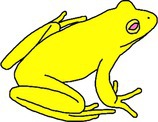	X (✓) I (✓) M (**↓**)	X (o) I (✓) M (**↓**)	X (o) I (o) M (**↓**)	Amelanism ‘Albino’ Complete lack of melanin pigment across all tissues Skin is yellow due to xanthophore pigment Retina red due to blood vessels Iris may be red but also with opaque/silver/white lustre due to iridophores	Allain and Goodman (2017)
Abnormal Frog A5	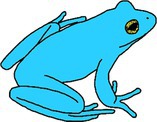	X (**↓**) I (✓) M (✓)	X (o) I (✓) M (✓)	X (o) I (o) M (✓)	Complete hypo‐xanthism (cutaneous) Potentially axanthism Skin is blue due to structural colouration from iridophores No iris abnormality	Nishioka and Ueda (1985a)
Abnormal Frog A6	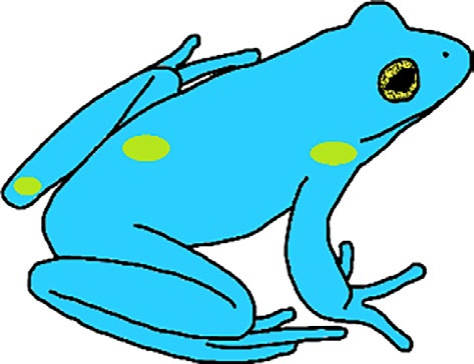	X (**↓**) I (✓) M (✓)	X (o) I (✓) M (✓)	X (o) I (o) M (✓)	Partial hypo‐xanthism (cutaneous) Small patches of normal green skin where xanthophore pigment is not affected No iris abnormality	Gould and McHenry (2024)
Abnormal Frog A7	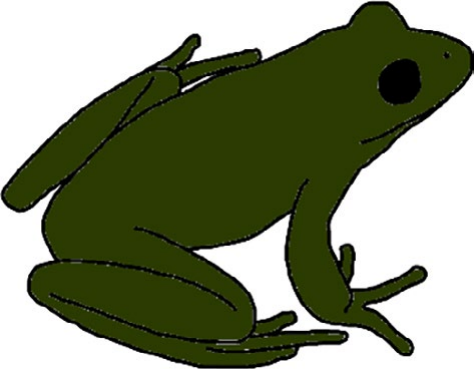	X (✓) I (**↓**) M (✓)	X (o) I (**↓**) M (✓)	X (o) I (o) M (✓)	Aniridism ‘Black eyed’ indicates iridophore deficiency Skin dark olive due to unaffected xanthophore and melanin pigments	Nishioka and Ueda (1985b)
Abnormal Frog A8	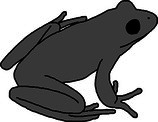	X (**↓**) I (**↓**) M (✓)	X (o) I (**↓**) M (✓)	X (o) I (o) M (✓)	Multi‐chromatophore deficiency Hypo‐iridism + xanism ‘Black eyed’ indicates iridophore deficiency Skin is dull grey from the presence of melanin	Richards et al. (1969)
Typical Frog B1	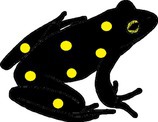	X (✓) I (o) M (✓)	X (o) I (✓) M (✓)	X (o) I (o) M (✓)	Black skin contains melanophores only Yellow patches high in xanthophores Iris gold iridescent	Lunghi et al. (2017)
Abnormal Frog B2	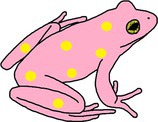	X (✓) I (o) M (**↓**)	X (o) I (✓) M (✓)	X (o) I (o) M (✓)	Complete hypo‐melanism (cutaneous) ‘Leucism’ Skin is semi‐translucent rather than white as iridophores are not present Skin is fleshy pink due to blood vessels No iris abnormality	Lunghi et al. (2017)
Abnormal Frog B3	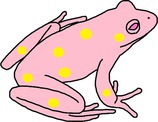	X (✓) I (o) M (**↓**)	X (o) I (✓) M (**↓**)	X (o) I (o) M (**↓**)	Amelanism ‘Albinism’ Complete absence of melanin across all tissues Skin semi‐translucent Tissues appear pink due to blood vessels	Lunghi et al. (2017)
Typical Frog C1	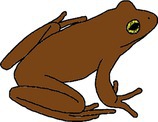	X (o) I (✓) M (✓)	X (o) I (✓) M (✓)	X (o) I (o) M (✓)	Skin is brown due primarily to melanin and opaque due to iridophores Iris gold iridescent	García‐Padrón and Bosch (2019)
Abnormal Frog C2	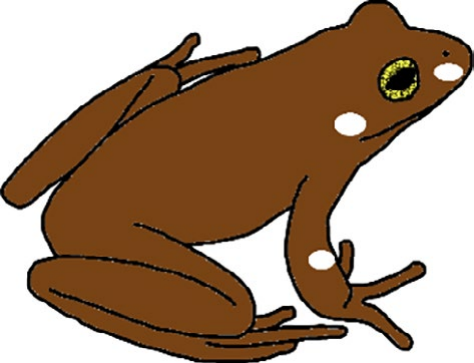	X (o) I (✓) M (**↓**)	X (o) I (✓) M (✓)	X (o) I (o) M (✓)	Partial hypo‐melanism (cutaneous) ‘Piebaldism’ Affected skin is white and opaque due to the presence of iridophores No iris abnormality	García‐Padrón and Bosch (2019)
Abnormal Frog C3	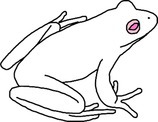	X (o) I (✓) M (**↓**)	X (o) I (✓) M (**↓**)	X (o) I (o) M (**↓**)	Amelanism ‘Albinism’ Skin is white and opaque due to the presence of iridophores Eye becomes red due to blood vessels	Few examples of truly white ‘albinos’ as small amounts of xanthophore causes light yellowing (e.g., Nishioka 1977)
Typical Frog D1	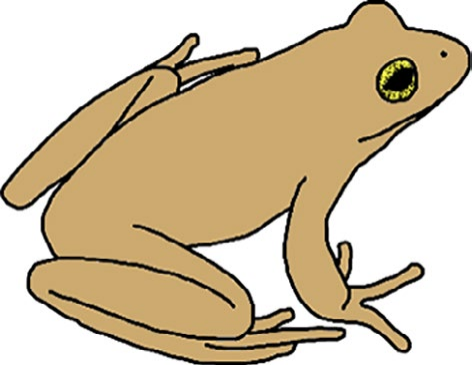	X (o) I (✓) M (✓)	X (o) I (✓) M (✓)	X (o) I (o) M (✓)	Skin beige due to the presence of iridophores and melanophores Iris gold iridescent	Turner (2017)
Abnormal Frog D2	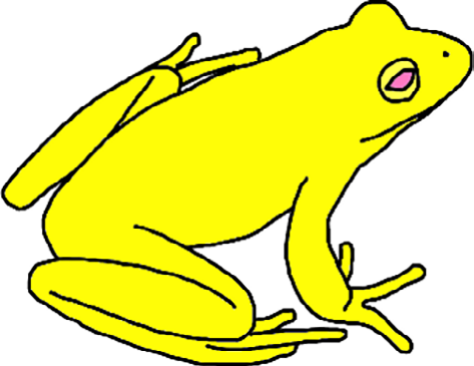	X (o) I (**↓**) M (**↓**)	X (o) I (**↓**) M (✓)	X (o) I (o) M (✓)	Multi‐chromatophore deficiency Hypo‐iridism/melanism ‘Black eyed’ and skin translucency indicates iridophore deficiency Skin is light in colour, pigmented by reduced melanin	Turner (2017)

*Note:* The occurrence of xanthophore pigment = X, iridophore reflecting platelets = I and melanophore pigment = M for each tissue type (skin, iris and retina) are provided, with their natural presence (✓), natural absence (o) and abnormal deficiency (↓) indicated.

## 2 Classification and Terminology

Chromatophore deficiencies are broadly classified based on the chromatophore types involved and whether an individual exhibits an abnormality anywhere across the body (e.g., Davis 2007). However, a more nuanced approach is to define the deficiency for each tissue type, thus allowing for separate tissue classifications. In this review, deficiencies across an entire tissue type will be referred to as ‘complete’. The term ‘partial’ when referring to chromatophore deficiencies can be ambiguous if it is used interchangeably to refer to either (i) a spatial deficiency (reduction/absence) in chromatophores or their colour‐producing content between different tissue areas/types or (ii) the global reduction in chromatophore cells/colour‐producing content but not their absence from tissue regions/areas where they naturally occur. For this review, the term ‘partial’ will be used when referring to spatial deficiencies in chromatophores within a given tissue type (e.g., deficient patches of skin). For example, an individual may broadly show deficiency in a chromatophore type but more specifically show partial deficiency in the skin but complete deficiency in the iris. Additionally, the deficiency may be permanent, which likely is caused by a genetic or developmental abnormality, or temporary, such as injury, exposure to pollutants or dietary deficit. This precludes the natural absence or variability in chromatophores observed within a population, which would not be considered an abnormality, or natural changes in chromatophore presence that occur during development, such as the delayed development of chromatophores until after specific life stages. Chromatophore deficiencies are referred to as forms of hypopigmentation and should not be confused with hyperpigmentation where there is an abnormal excess of these cells/pigments.

## 3 Hypo‐Melanism—Deficiency in Melanophores

The melanin pigment within melanophores absorbs light across a broad range of wavelengths and typically gives tissues a dark colour, ranging from black to brown or even grey when melanin is less concentrated (McGraw 2006). A deficiency in melanin, which may be caused by a reduction, abnormality or complete absence in melanophores and/or their melanin pigment, results in forms of hypo‐melanism affecting the skin, iris and/or retina (Table 2). Past reviews in other animal groups have separated hypo‐melanism from amelanism (e.g., Lucati and López‐Baucells 2017); however, I have categorised amelanism as an extreme type of hypo‐melanism, given that it makes practical sense to incorporate all melanin deficiencies under one broad umbrella term (Table 2).

**TABLE 2:** Classification of chromatophore deficiencies, including hypo‐melanism, hypo‐xanthism and hypo‐iridism among amphibians.

Deficiency	Types	Definition	Tissue‐types	Tissue subtype	Definition	Persistence	Other terms
Hypo‐melanism	Non‐amelanism	Deficiency/abnormality in melanophore cells or pigment in at least one tissue. However, melanin is still present in some tissues.	Ocular (iris) Ocular (retinal) Cutaneous	Complete	Melanin absent or present in low amounts across the tissue	Permanent Temporal	Leucism Partial albinism Partial amelanism
				Partial	Deficiency in melanophore cells or pigment in some parts of the tissue		Leucism Partial albinism Partial amelanism Piebaldism
	Amelanism	Total absence of melanin pigment across all tissues	All tissues	None	—		Pure/complete albinism
Hypo‐xanthism	Non‐axanthism	Deficiency/abnormality in xanthophore cells or pigment in at least one tissue. However, xanthophore pigment still present in some tissues.	Ocular (iris) Cutaneous	Complete	Xanthophore pigment absent or present in low amounts across the tissue	Permanent Temporal	None
				Partial	Deficiency in xanthophore cells or pigment in some parts of the tissue		None
	Axanthism	Total absence of xanthophore pigment across all tissues	All tissues	None	—		None
Hypo‐iridism	Non‐aniridism	Deficiency/abnormality in iridophore cells or reflecting platelets in at least one tissue. However, platelets are still present in some tissues.	Ocular (iris) Cutaneous	Complete	Iridophore reflecting platelets absent or present in low amounts across the tissue	Permanent Temporal	Black‐eyed
				Partial	Deficiency in iridophore cells or reflecting platelets in some parts of the target tissue		Black‐eyed
	Aniridism	Total absence of iridophore reflecting platelets across all tissues	All tissues	None	—		Black‐eyed

### 3.1 The Connection Between Amelanism and Albinism

Amelanism is an extreme form of hypo‐melanism where there is a total lack of melanin across all tissues. It has been commonly referred to as albinism (Davis 2007; Lucati and López‐Baucells 2017), although albinism has been used to refer to a lack of pigment in general (e.g., Acevedo et al. 2009). Amelanism is typically caused by the inheritance of mutated gene variants, resulting in the lack of biosynthesis of melanin pigment across the skin and eyes (iris and retina) (Lunghi et al. 2017; Sazima 1974). This causes melanophore cells to still be present but for their melanosomes to possess no melanin pigment (Taylor and Bagnara 1972). A defining feature of amelanism is the lack of melanin pigment in the retinal pigment epithelium (RPE) cells of the retina (Sazima 1974). The RPE occurs at the back of the eye and typically gives the pupil a black appearance, with melanin absence causing the pupil to instead appear pink/red or black with a red sheen due to reflection from capillaries that would otherwise be masked by the melanin pigment (Sazima 1974). Thus, amelanism can be easily distinguished from other forms of hypo‐melanism if the retina is totally deficient in melanin but only if melanin is simultaneously absent in the skin and iris. The iris of amelanistic individuals may also have a red shine in the absence of melanin but can still be opaque and white/silver given the unaffected presence of iridophores (e.g., Westaway 2025).

There are types of melanin deficiencies that may result in a similar appearance and be confused with amelanism. This includes a condition that results in a lack of retinal pigmentation (e.g., Smith‐Gill et al. 1972), which would more correctly be defined as complete hypo‐melanism of the retina but not true amelanism as melanin pigment is still present in other tissues. Additionally, there is a mutation referred to as periodic albinism where melanin synthesis is not inhibited completely as affected individuals gain melanin at the larval stage but abnormal depigmentation occurs after metamorphosis (Eagleson et al. 2010; Hoperskaya 1975), which would be more correctly defined as temporal amelanism but dependent on whether any melanin pigment remains at the stage where depigmentation occurs. Any instance of melanin production across life stages or tissue types precludes the condition from being permanent amelanism.

### 3.2 Hypo‐Melanism and Leucism as Related Terms

A more ambiguous term that has been associated with hypo‐melanism is leucism, which broadly occurs when there is depigmentation of tissues such as the skin and perhaps the iris (but not the retina). However, the exact definition of leucism varies between studies, including: (1) depigmentation (or lack of pigmentation) in general with no specificity of the types of chromatophores affected (Turner 2017; Moore and Ouellet 2014), or (2) any deficiency which results in the expression of white (as the name implies; luecos = white) (Konter 2015; Lucati and López‐Baucells 2017) or (3) partial albinism and specifically related to a deficiency in melanin (Davis 2007; Abreu et al. 2013). Ambiguity exists in the use of this term as it has commonly been applied to animals where melanin is the primary pigment type (Lucati and López‐Baucells 2017), resulting in any reference to depigmentation to be automatically and only the result of melanin deficiency. This has led to confusion, as highlighted by previous researchers (e.g., Davis 2007), as leucism has often been defined by the appearance of the affected tissue (i.e., white) rather than the specific pigments/cells involved (Lucati and López‐Baucells 2017). In contrast to albinism, leucism has been related to the failed migration of chromatophores and/or their reduced production during embryonic development (Lucati and López‐Baucells 2017).

If the first definition of leucism is used, then it could apply to the deficiency in any combination of chromatophores, so long as all chromatophores present are deficient. If the second definition is used, it could apply to any combination of chromatophore deficiencies, so long as the resultant colour expression is white. Either of these definitions are highly ambiguous and confusing, as the chromatophores affected would theoretically differ between species and even skin sections of the same individual that have varying chromatophore compositions. Abiding by either of these definitions, the term leucism has been applied incorrectly in some amphibian studies where there was an observed deficiency in one type of chromatophore but not also the co‐occurring chromatophore, and the resultant colour expression was not white (e.g., Brito‐Zapata 2021). The third definition would appear to be less ambiguous, particularly as it was originally applied to animals where depigmentation can only relate to a deficiency in melanin, whereby leucism would be defined as any form of hypo‐melanism excluding amelanism. This definition would be beneficial when exploring another ambiguous term referred to as piebaldsim, which relates to depigmentation of some sections of tissues, such as skin blotches (Acevedo et al. 2009). If we were to treat piebaldsim as a form of partial leucism, as suggested by Neff et al. (2015), and specifically relate it to a reduction/absence of melanin in localised areas of tissues, then it would be aligned with the definition of partial hypo‐melanism of the affected tissue.

Within the amphibian literature, this ambiguity in the meaning of leucism is apparent, which has been defined by some as a global reduction in pigment or total depigmentation, with no mention of whether depigmentation can involve any or all chromatophore types or melanophores specifically (de Lima Moraes and Kaefer 2015; García‐Padrón and Bosch 2019; Rivera‐Prieto and Marín 2017). Most observations of leucism and piebaldism among amphibians likely pertain to a deficiency in melanin specifically (hypo‐melanism). I suggest that the terms albinism, leucism and piebaldism should be used as categories that specifically and only encompass the different patterns of melanin‐based deficiencies (Bechtel 1995). Given their ambiguous application thus far, however, the terms may be better off retired so as to prevent any future issues. However, I have included these ambiguous terms when they have been used by the authors of case studies presented below, to show how they relate to the terms hypo‐melanism and amelanism.

### 3.3 Leucism and Ocular Depigmentation

A defining feature of leucism is the absence of depigmentation of the retina, which is specifically related to melanin as this is the only pigment type present in the RPE. Leucism does not affect the retina and so the pupil retains a normal black colouration (de Lima Moraes and Kaefer 2015), which is how it can be easily differentiated from amelanism (true albinism). This difference in melanin presence between these regions occurs as melanin pigment cells in the skin and iris are derived from the neural crest (Du Shane 1935; Rubin et al. 1986; Sturm and Larsson 2009), while RPE cells of the retina originate from the neuroectodermal sheath of the eyecup (Lamoreux et al. 2010; Schraermeyer and Heimann 1999; Sturm and Larsson 2009). Referring to leucism as a deficiency in melanin would have the benefit of aligning the depigmentation seen in non‐retinal tissues to the lack of depigmentation in retinal tissue.

Ambiguity exists in the literature with regard to whether the iris can be impacted by leucism in addition to skin, which would be expected given that melanophores for both regions have the same embryonic origin (Sturm and Larsson 2009). Indeed, several authors suggest leucism in amphibians does not involve melanin deficiency in the iris (de Lima Moraes and Kaefer 2015; Lunghi et al. 2017; Moore and Ouellet 2014), which is also suggested by authors referring to other animal groups (e.g., Van Grouw 2013). However, these authors do not explicitly state the effect of melanin deficiency for both the iris and retina as separate parts of the eye. It is, however, surprising that there are few records of leucism (hypo‐melanism) among amphibians involving melanin deficiency within the iris. An exception is the absence of normal iris pigmentation (one or both eyes) recorded in the moor frog, *Rana arvalis* (Vershinin 2004). While the author states it is as a form of partial albinism in *R. arvalis*, this is difficult to assess given they do not allude to the colour of the retina or other tissues; it would be more accurately defined as partial or complete hypo‐melanism of the iris tissue dependent on whether one or both eyes are affected. Despite the lack of evidence among amphibians, some hereditary abnormalities among animals in general provide evidence that leucism can cause an absence of melanin for both the skin and iris (Pingault et al. 2010). For example, Waardenburg–Klein syndrome in humans results in melanin deficiency in the hair, causing white patches (partial hypo‐melanism of the skin/hair), as well as blue eyes (complete hypo‐melanism of the iris) caused by an absence of melanin in this tissue (Pingault et al. 2010). There is a need for more exploration of how the migration of pigment cells from the neural crest during embryonic development differs between the skin and iris and how this may cause one to be pigmented with melanophores and not the other.

### 3.4 Colour Expressions With Hypo‐Melanism

Even if there is a deficiency in melanophores, amphibians may not necessarily express the stereotypical white observed in other animal types (van Grouw 2006), which is most apparent in amelanistic individuals. This is due to the presence of other chromatophore types that are present and not affected by the abnormality (Gould and McHenry 2024; Nishioka and Ueda 1985a; Smith‐Gill et al. 1972).

### 3.5 Case Study: Hypo‐Melanism Causing Skin Yellowing

Green skin typically forms in the presence of all three dermal chromatophore types (Bagnara and Hadley 1973; Nielsen and Dyck 1978; Grether et al. 2004). In particular, the iridophores found in green skin possess highly ordered platelets that selectively reflect blue and green wavelengths of incoming light, while all other wavelengths are transmitted to the deeper melanophore layer and absorbed (Nielsen and Dyck 1978). However, the blue and green wavelengths reflected from the iridophore layer must pass through the overlying xanthophore layer, which selectively filters the blue portion, allowing only the green wavelengths to leave the skin in addition to yellow reflectance from the xanthophore to produce a yellow–green colour (Nielsen and Dyck 1978). As such, truly green wavelengths are being perceived by the viewer; it is not due to additive colour mixing whereby blue light reflected from the iridophore and yellow reflection from the xanthophore are perceived as green by the observer.

**FIGURE 1:**
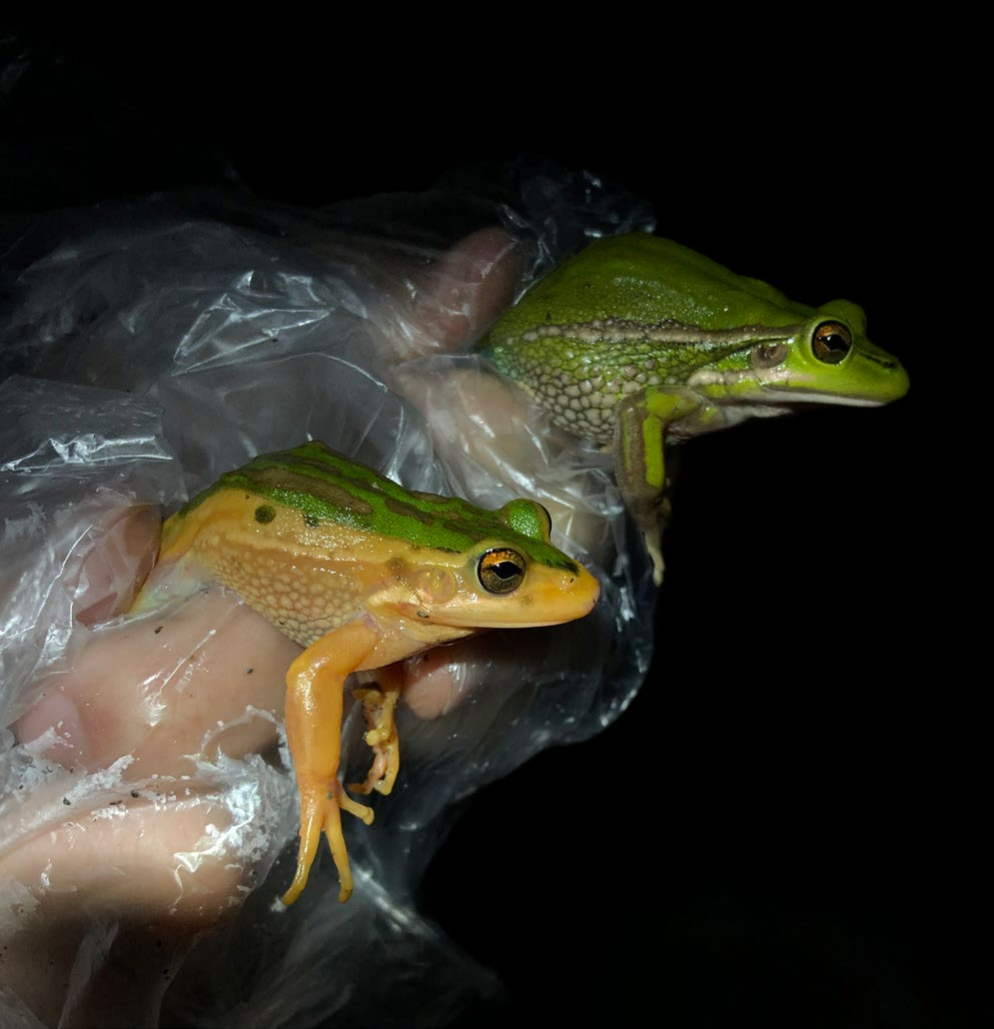
Skin yellowing in an adult green and golden bell frog, *Litoria aurea* , caused by hypo‐melanism. The abnormal individual (left) is partially hypo‐melanistic (cutaneous) or ‘piebald’ and shows patches of yellow dorsal skin that should be green. A typical individual (right) is shown as a direct in‐photo comparison.

A deficiency in melanophores in typically green skin results in the expression of yellow instead, given the unaffected presence of xanthophores (which provide the yellow pigment) and iridophores from the dermis and lower layers below the melanophores that are revealed in the absence of melanin (Nielsen and Dyck 1978). These lower layered iridophores scatter light and dampen the structural colour produced by the more orderly dermal iridophores, thus producing white light that brightens the xanthophore pigment (Forbes et al. 1973). For example, the green and golden bell frog, *Litoria aurea* , typically presents with a pea‐green dorsum, but individuals have been recorded with abnormal patches of dorsum yellowing, which is likely the result of partial hypo‐melanism (piebaldism) of the skin as opposed to amelanism (albinism) given the lack of retinal depigmentation (Gould and McHenry 2024; Figure 1).

A unique exception to this typically yellowing seen in green frogs with hypo‐melanism is found in species such as Schlegel's tree frog, *Rhacophorus schlegelii* , which naturally lose skin melanophores as they mature (Nishioka and Ueda 1985a). In these species, the melanophores are naturally replaced by violeophores, a rarer, dark‐violet chromatophore derived from xanthophores, which effectively complete the dermal chromatophore unit and result in green skin. Thus, even amelanistic (albino) individuals still show green skin while the eyes are the stereotypical red (Nishioka and Ueda 1985a). Yellow skin abnormalities thus only form in these species if there is an abnormal deficiency in violeophores, leaving only the presence of unaffected xanthophores and iridophores in the skin (Nishioka and Ueda 1985b). These yellow aberrations would effectively be termed hypo‐violeothic (‘leucistic’).

Brown/dark brown/dark olive skin can naturally form when xanthophores and melanophores are present together in the skin as a combination (Forbes et al. 1973; Nishioka and Ueda 1985b). A deficiency in melanophores within such skin sections will cause the predominance of the xanthophore pigment (de Lima Moraes and Kaefer 2015). For example, hypo‐melanism has been recorded in the epibatidine poison frog, *Epipedobates anthonyi* (Brito‐Zapata 2021). Typically, this species has a very dark brown/black dorsal and ventral base with white stripes. Partial hypo‐melanism of the skin (piebaldism) was found in an individual with (i) an abnormally yellow dorsal surface with brown patches, indicating the presence of unaffected xanthophores to give the yellow colour, with some areas where melanin was unaffected, (ii) the maintenance of white stripes, indicating the presence of unaffected iridophores in these regions, (iii) a semi‐translucent ventral surface, indicating a lack of melanin causing translucency given no other chromatophores are naturally present in this region and (iv) irises that had a golden lustre, indicating not effect on iridophores, and black retina, indicating that it was not amelanism (albinism).

### 3.6 Case Study: Hypo‐Melanism Causing Skin Translucency and Paleness

For species with black skin, a melanophore deficiency typically leads to the expression of grey skin if some melanin remains or white skin if mostly absent, but only if iridophores are also present and unaffected (Baker and Biddle 2020; García‐Padrón and Bosch 2019; Neff et al. 2015). In contrast, the skin becomes pink/semi‐translucent if no other chromatophore types are present (Lunghi et al. 2017; Rivera‐Prieto and Marín 2017). These abnormal colour expressions can only occur if the skin is naturally absent in xanthophores, which would otherwise lead to the expression of yellow skin instead (Gould and McHenry 2024).

For example, fire salamanders, *Salamandra Salamandra* , typically have a black skin base that is melanin rich, with yellow patches (Lunghi et al. 2017). Albino (amelanistic) and leucistic (complete hypo‐melanism of the skin) individuals of this species both express semitranslucent pink skin, which must occur as there are no iridophores or xanthophores naturally present, but continue to express yellow patches, which must be due to the presence of unaffected xanthophores in these specific areas (Lunghi et al. 2017). Albino (amelanistic) individuals showed red eyes, though the authors are not specific on the colouration of the iris and retina separately, while the eye colour of leucistic (complete hypo‐melanistic skin) individuals is not specifically mentioned but appears normally pigmented in images (Lunghi et al. 2017). Similarly, leucism (complete hypo‐melanism of the skin) has been recorded in the yellow‐striped poison frog, *Dendrobates truncatus*, which typically has a dark brown/black skin base with white/yellow stripes and dark irises (Rivera‐Prieto and Marín 2017). A leucistic (complete hypo‐melanism of the skin) individual presented with the typical white/yellow striping, indicating the presence of unaffected xanthophores and iridophores, while the skin base was translucent‐pink, indicating a deficiency of melanophores while no other chromatophore types were naturally present, and the iris and retina remained dark, indicating no effect on their melanin pigment and that it was not amelanism.

### 3.7 Ambiguous Cases

In some species, the presence of xanthophores and melanophores within the same skin region together can make a visual determination of hypopigmentation challenging. For example, a leucistic (complete hypo‐melanism of the skin) Stephen's rocket frog, *Anomaloglossus stepheni* , presented with (i) opaque white rather than the typical dark skin spotting, indicating the retention of unaffected iridophores but loss of melanin, (ii) absence of typical dark brown banding, indicating the loss of melanin and (iii) unaffected eyes, both iris and retina, indicating it is not amelanism (albinism) (de Lima Moraes and Kaefer 2015). Yet, the species retained the typical opaque, ochre dorsum base colour, which could be confused with the retention of melanin but is more likely caused by the unaffected presence of xanthophores given the orange colour, as suggested by the authors. Additionally, some species such as Zeus' robber frog, *Eleutherodactylus (Syrrophus) zeus,* have complex skin colouration that includes a purple‐brown dorsal base with blackish and yellow mottling. A partially leucistic ‘piebald’ (partial hypo‐melanism of the skin) individual was recorded with white skin patches in areas which were primarily brown, which likely occurred due to a deficiency in melanophores but the unaffected presence of iridophores in these areas, while the iris and retina colouration were unaffected (the latter indicating it is not amelanism) (García‐Padrón and Bosch 2019). It is difficult to assess if there is an abnormality in melanin alone or a co‐occurring deficiency in xanthophore pigment in this case.

### 3.8 Hypo‐Melanism Versus Xanthochromism

The expression of yellow skin due to the presence of unaffected xanthophores in skin regions with a melanophore deficiency can be confused with another abnormality referred to as xanthochromism. The latter is an ambiguous term that is suggested to be caused either by (i) a multi‐chromatophore abnormality where there are higher concentrations or an atypical predominance of yellow pigments in xanthophores and a simultaneous deficiency in melanophores (Allain et al. 2023; Cotts, Prestes, et al. 2023; Cotts, Slifkin, et al. 2023) or (ii) simply the unmasking of xanthophores when there is a melanin deficiency (Mitchell 1994; Wakida‐Kusunoki et al. 2024). I do not believe that the latter should be defined as xanthochromism, as there is no change in xanthophore expression that creates the yellow colouration, merely a deficiency in melanophores alone. It may be difficult to discern whether an abnormal yellow individual is the result of hypo‐melanism or xanthochromism, except the latter may cause an excessive vibrancy in areas of skin that are normally yellow or a yellow tinge across much of the body (Allain et al. 2023).

A complex case was recorded by Allain and Goodman (2017) in the common frog, *Rana temporaria* , which typically has a light to dark brown dorsal colour but an individual was found with opaque yellow skin, suggesting a deficiency in melanin across all skin regions but the unaffected presence of xanthophores and iridophores. The individual also presented within a red shine to the eyes, which would suggest amelanism (albinism), yet the authors suggest it is a case of xanthochromism. Allain and Goodman (2017) allude to xanthochromism being characterised by a lack of skin pigment except for xanthophores, which is in effect simply a case of melanin deficiency. The term xanthochromism should not be used in this case as it does not allude to the chromatophore type that is affected so much as the colour the forms via the unmasking of the prevailing chromatophores (namely xanthophores). This is in contrast to cases where there may truly be an abnormal increase in xanthophore‐like pigment cells (e.g., erythrophores) in addition to a deficiency in melanophores (hypo‐melanism) (e.g., West and Allain 2020).

## 4 Hypo‐Xanthism: Deficiency in Xanthophores

Xanthophores impart colours to skin and the iris ranging from yellow to orange and red (Bagnara 1966; Bagnara and Hadley 1973): I will refer to xanthophore as primarily imparting yellow to avoid confusion. An abnormal deficiency in xanthophores or their yellow pigment content (pterinosomes and/or carotenoids) is referred to as hypo‐xanthism, with the complete lack of xanthophore pigment across all tissue types referred to as axanthism (Table 2). This deficiency in xanthophores leads to a decrease in the filtering of shorter wavelengths of light leaving the skin and/or iris (Bagnara et al. 1978; Bagnara and Hadley 1973; Berns and Narayan 1970; Nishioka 1977), with the resulting colour expression dependent on the extent of the deficiency and whether other types of chromatophores are present yet unaffected (Nishioka and Hiroaki 1985).

### 4.1 Case Study: Hypo‐Xanthism in Typically Green Skin

For skin regions that are typically green, which requires the presence of xanthophores, iridophores and melanophores (Nielsen and Dyck 1978), hypo‐xanthism results in the expression of a blue colour instead, given the presence of iridophores and melanophores that are unaffected (Berns and Narayan 1970; Miller et al. 2018; Nishioka 1977; Nishioka and Ueda 1985a). The skin becomes blue as (i) the iridophores possess highly ordered platelets that reflect green/blue wavelengths and the blue wavelengths are now able to leave the skin without being filtered by the xanthophores, (ii) non‐blue/green wavelengths transmitted through the iridophore layer are absorbed by the deeper melanophore layer and prevented from being scattered by deeper, reflective skin layers (Bagnara and Hadley 1973; Berns and Narayan 1970; Nielsen and Dyck 1978).

**FIGURE 2:**
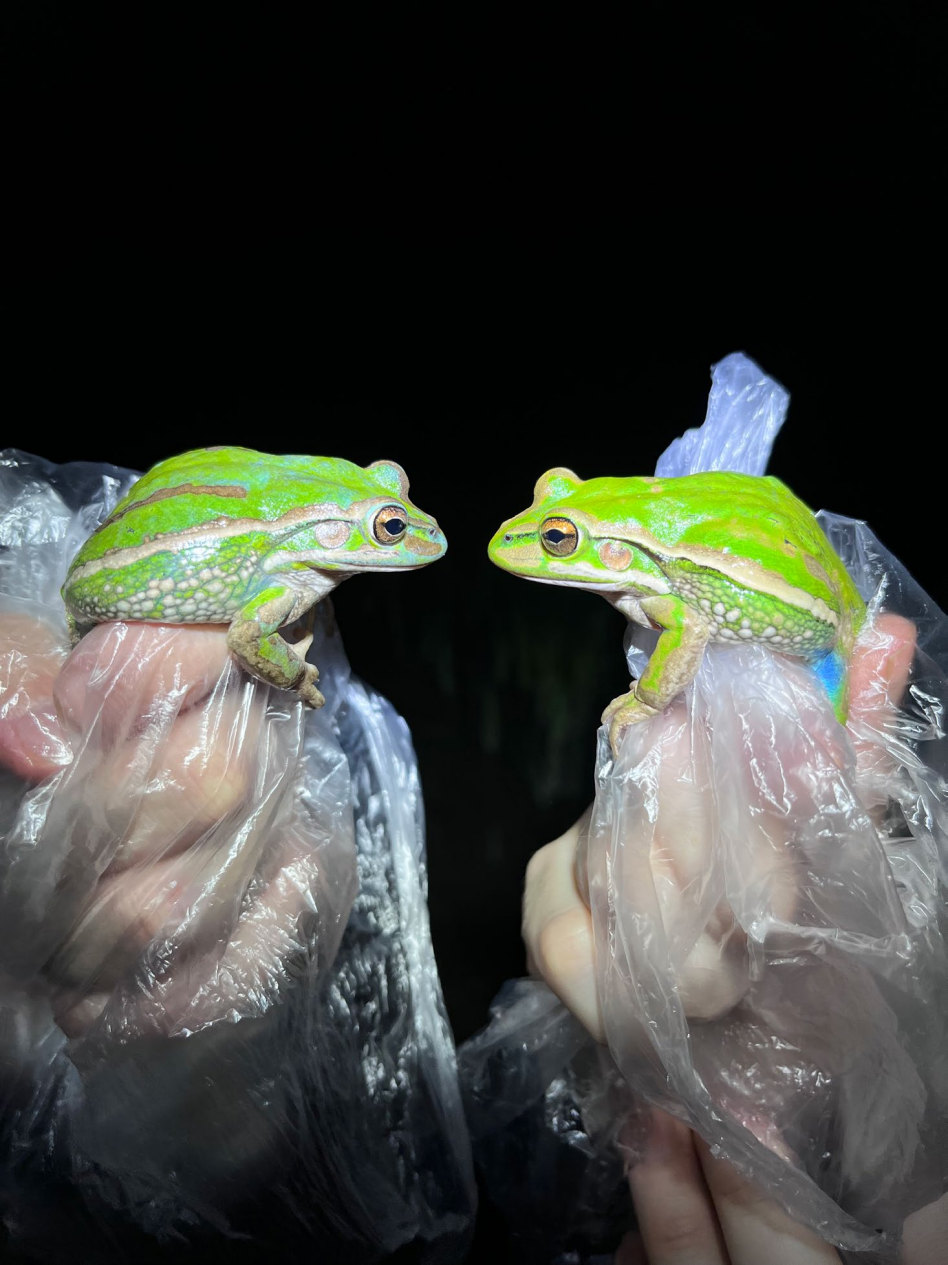
Skin blueing in an adult green and golden bell frog, *Litoria aurea* , caused by hypo‐xanthism. The abnormal individual (left) is partially hypo‐xanthic (cutaneous) and shows patches of bluish‐green dorsal skin that should be green. A typical individual (right) is shown as a direct in‐photo comparison.

**FIGURE 3:**
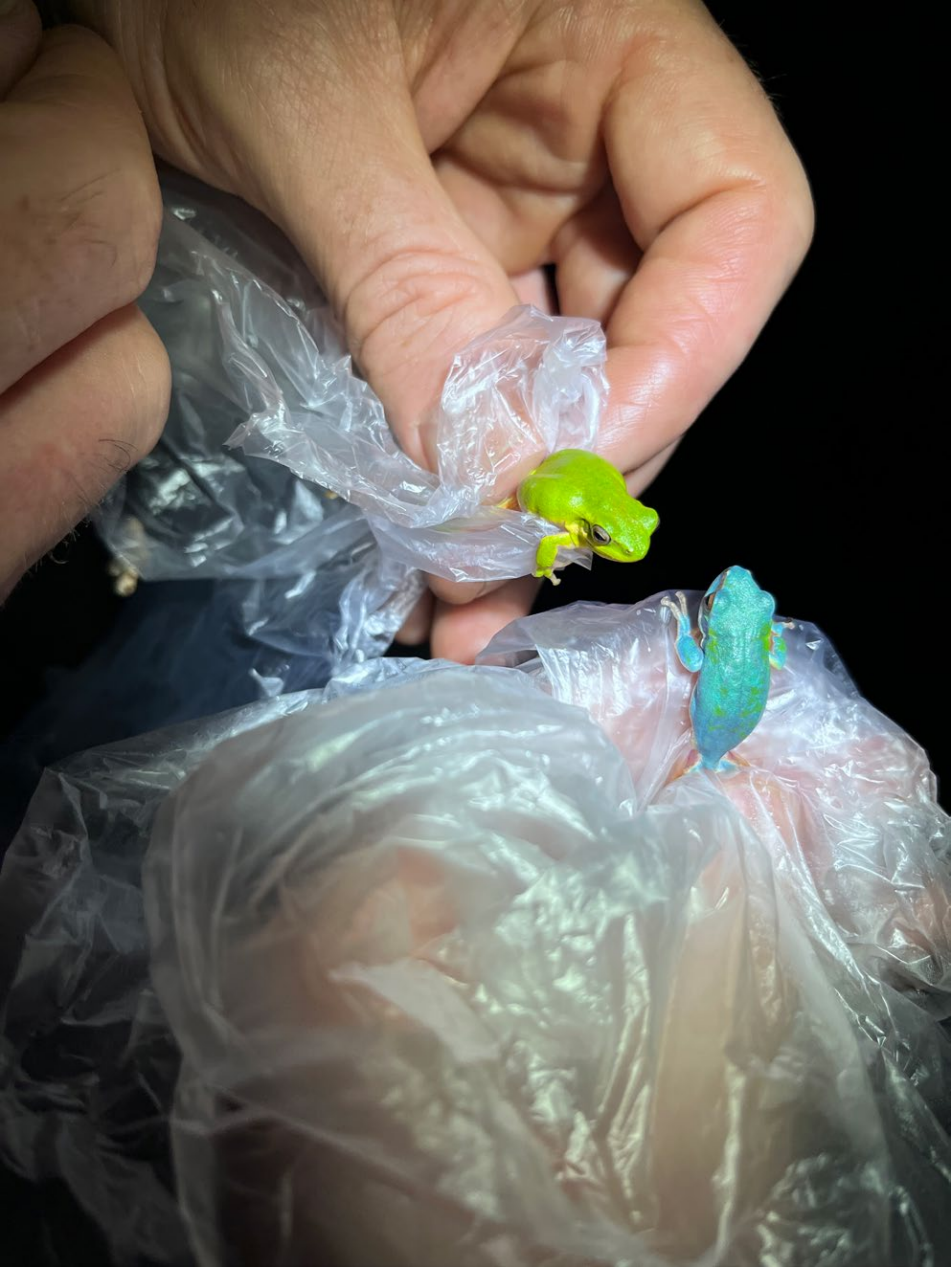
Skin blueing in an adult eastern sedge frog, *Litoria fallax* , caused by hypo‐xanthism. The abnormal individual (right) is nearly completely hypo‐xanthic (cutaneous) and shows blue dorsal skin that should be green. A typical individual (left) is shown as a direct in‐photo comparison.

Cases have been recorded where there is likely only a reduction but not total absence in xanthophores and/or xanthophore pigment, resulting in typically green skin being a blue–green as opposed to blue, such as a partially hypo‐xanthic *L. aurea* adult recorded by Gould and McHenry (2024) (Figure 2). In other cases, there is a true bluing of typically green dorsal skin (e.g., *Litoria fallax* ; Figure 3), suggesting a complete absence of xanthophore pigment in affected skin (Gould and McHenry 2024). It is difficult to determine by visual assessment alone whether such cases are true forms of axanthism where there is no xanthophore pigment at all (e.g., Nishioka and Ueda 1985a) or simply severe cases of complete hypo‐xanthism of the skin, but a stark blue skin colouration and absence of any green hue would suggest axanthism so long as there is also an absence of xanthophore pigment in the iris. Ambiguous cases occur where only small amounts of normal green skin colouration remain (Figure 3), and whether these abnormal individuals should be defined as partial or complete hypo‐xanthics (Gould and McHenry 2024); they should not be defined as axanthic if any xanthophore pigment is present. Additionally, Nishioka and Ueda (1985b) have recorded temporal axanthism in the Japanese tree frog, *H. japonica* , whereby individuals that were entirely blue developed green spots later in adulthood, indicating the emergence of xanthophore pigment to some extent and their transition to showing partially hypo‐xanthic skin.

### 4.2 Case Study: Hypo‐Xanthism in Typically Non‐Green Skin

For species with only xanthophores and melanophores present in skin regions, which may result in the expression of typically brown or dark olive colours, a deficiency in xanthophores will result in a darkening of the skin and predominance of the brown/black melanin pigment of the melanophores only (Jablonski et al. 2014). In cases where xanthophores typically co‐occur with iridophores without melanophores present, the iridophore typically brightens the colour expressed by the xanthophore pigment (Forbes et al. 1973). A total lack of xanthophore pigment (axanthism) in this particular case would result in the skin no longer expressing colour and instead becoming pale and white given the scattering of light from the iridophores alone (Forbes et al. 1973).

### 4.3 Misinterpretations of Hypo‐Xanthism

Ambiguity exists in the literature as some authors suggest hypo‐xanthism in amphibians is not only a deficiency in xanthophores alone but also in cases where there is a deficiency in both xanthophores and iridophores together. For example, Jablonski et al. (2014) separate hypo‐xanthism (although they use the term axanthism) into three main expressions: (i) blue colouration, (ii) darkening of body colouration, (iii) blackening of the eyes. Only the first of these categories is specifically related to a deficiency in xanthophores, while the other two categories are related to the effect of an iridophore deficiency. I suggest this should be avoided, as deficiencies in iridophores result in an entirely different suite of changes in skin and eye colour expression, and iridophore deficiencies may occur on their own. Their effects should be discerned when possible, as this may also lead to confusion, as non‐experts may attribute colour abnormalities that are specifically the result of iridophores defects as being hypo‐xanthic. Additionally, it entirely excludes cases where a deficiency in xanthophores may also co‐occur with abnormal changes in melanophores (Nishioka and Ueda 1985b). There is a need for consistency and for hypo‐xanthism to be (i) specifically related to a deficiency in xanthophores and (ii) used in the description of any abnormality where there is a deficiency in xanthophores, even if other chromatophores are affected or not.

### 4.4 Deficiency in Pterinosomes Versus Carotenoids

Along with pteridines, carotenoids within xanthophores and erythrophores play a critical role in colour expression (Bagnara and Hadley 1973). These pigments are typically interspersed between pteridines when they are present and absorb short wavelengths much like pteridines, imparting yellow, orange or red colouration based on the types of carotenoids that are present within these cells (Brenes‐Soto and Dierenfeld 2014; Hama 1963). Carotenoids cannot be synthesised by chromatophores (Bagnara 1998) and are instead acquired from food. Thus, animal condition and diet can influence the visual appearance of the skin based on carotenoid types consumed and their concentration within chromatophores. For example, Matsui et al. (2002) found that wild‐caught adult Japanese newts, *Cynops pyrrhogaster* , had high amounts of carotenoids in xanthophores/erythrophores present within ventral skin, resulting in a red colouration, suggesting that carotenoids are the dominant pigment type in these cells that imparts red colouration as opposed to pteridines, which are lost at metamorphosis (Obika 1963). In contrast, laboratory‐kept *C. pyrrhogaster* individuals possessed yellow as opposed to red skin because of a carotenoid‐deficient diet leading to less of this pigment within these cells; in this species, the skin contains riboflavin that in effect may become a substitute for pteridines that imparts a yellow colouration within the xanthophores (Matsui et al. 2002). It remains to be debated whether a dietary deficit in carotenoids should be considered hypo‐xanthism if there is no actual abnormality or deficiency in xanthophore cells or pterinosome pigment biosynthesis, unless the dietary deficit is caused by unnatural circumstances (e.g., captivity).

## 5 Hypo‐Iridism: Deficiency in Iridophores

Iridophores produce structural colours or white reflectance dependent on the orientation of the reflecting platelets (Bagnara 1983; Bagnara et al. 1969; Kobelt and Linsenmair 1986; Nielsen and Dyck 1978). Typically, the platelets of dermal iridophores are highly ordered in green skin and play a critical role in the production of green, while disordered platelets typically give rise to white and are often found in the ventral skin of amphibians. Given their role in light reflection, a deficiency in iridophores, which is referred to as hypo‐iridism, typically results in (i) darkening of the skin and (ii) an increase in skin translucency (Nishioka and Ueda 1985b). The effect is dependent on the extent of the iridophore deficiency, including whether reflecting platelets of these cells remain present, are abnormal or totally absent, or whether iridophores are entirely absent, as well as the presence of co‐occurring chromatophores (Nishioka 1977; Nishioka and Ueda 1985b). If reflecting platelets are completely absent across all tissues, then the deficiency should be referred to as aniridism (Table 2). The effect of an iridophore deficiency on the retinal tapetum is not explored here but may form part of a diagnosis (e.g., lack of eyeshine) with future clarification of the cells that make up this retinal layer, their origin and how these are related to iridophores (Oliphant et al. 1992).

### 5.1 Partial Hypo‐Iridism

Partial hypo‐iridism has been recorded in some species, such as mutants of *H. japonica* , where iridophores remain present in some portions of the iris to give it a typical golden lustre in some locations (Nishioka and Ueda 1985b), or cases when the iris is black due to the absence of iridophores (complete hypo‐iridism of the iris), but iridophores are still normal and present within the skin (Richards and Nace 1983). In these cases, islands of iridophores are present at possibly normal levels and surrounded by a landscape where they are deficient, indicating that they are not true cases of aniridism. The deficiency of iridophores specifically in the eyes and not the skin detected by Richards and Nace (1983) provides evidence that the migration of pigment cells to the iris may differ from that of the skin and explains the ambiguity that exists around leucism and its impact on the iris.

### 5.2 Case Study: Hypo‐Iridism Causing Skin Darkening

Skin darkening due to an iridophore deficiency occurs specifically in regions where melanophores are also present yet unaffected (Richards and Nace 1983), with the melanin pigment subsequently allowed to absorb more light and become the predominant colour that is expressed. This darkening is also apparent in the iris, which typically has a metallic gold/silver shimmer or lustre if iridophores are present but becomes a dark brown/black when absent as the underlying melanophores are revealed or predominate (Nishioka and Ueda 1985b). Individuals with this iris abnormality are often referred to black‐eyed variants or mutants (Nishioka and Ueda 1985b) or dark pigment variants (Richards and Nace 1983). It is important to note that black‐eyed variants can occur across all types of chromatophore abnormalities, but it must involve an iridophore deficiency.

### 5.3 Case Study: Hypo‐Iridism Causing Skin Translucency

**FIGURE 4:**
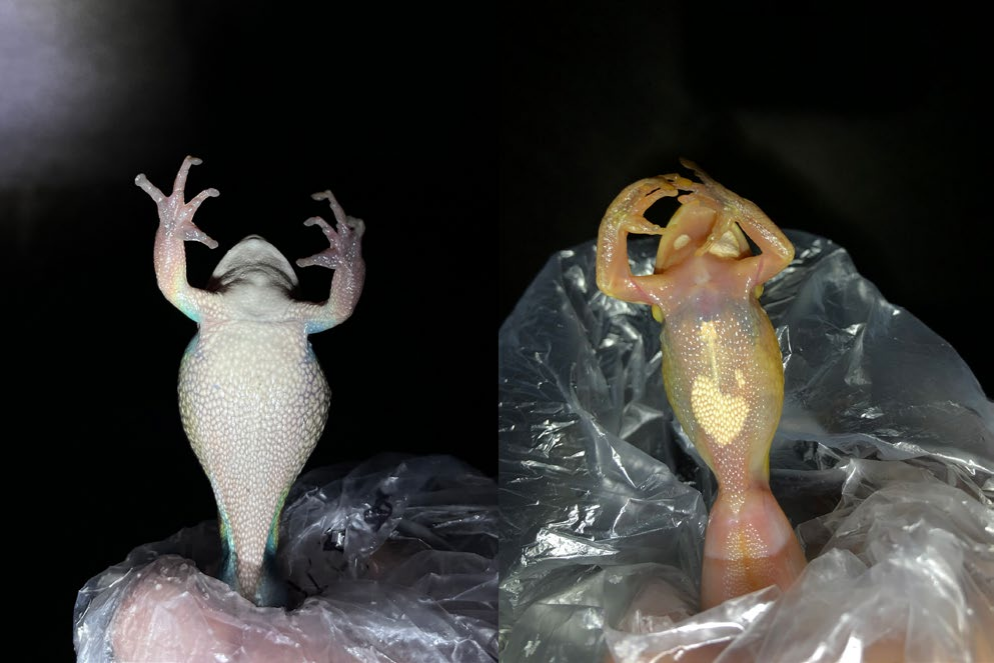
Skin translucency in an adult green and golden bell frog, *Litoria aurea* , caused by hypo‐iridism. The abnormal individual (right) is partially hypo‐iridic (cutaneous) and shows regions of semi‐translucent ventral skin that should be opaque white. A typical individual (left) is shown as a direct in‐photo comparison.

Skin translucency is a hallmark of iridophore deficiency and is particularly apparent in regions of skin where no other chromatophores are naturally present (Nishioka 1977; Nishioka and Ueda 1985a, 1985b). Skin regions are typically opaque and white when iridophores are present alone (Richards and Nace 1983), which often occurs across ventral surfaces of amphibians (Frost et al. 1984; Fukuzawa et al. 1995), but becomes semi‐translucent when there is an iridophore deficiency as light is no longer being reflected by these cells but allowed to transmit through the skin into the body cavity (Nishioka 1977; Nishioka and Ueda 1985a, 1985b). In these circumstances, full transparency does not occur as light scattering is still produced by collagen fibres present in the skin (Nishioka 1977; Nishioka and Ueda 1985b), which can make it difficult to assess whether an individual is abnormally white or abnormally see‐through or somewhere in between (e.g., Westaway 2025). Additionally, the skin may often have a fleshy pink hue due to light reflecting from blood capillaries beneath the dermis within the hypodermis that are now exposed (Richards and Nace 1983). For example, adult *L. aurea* have been found with abnormal regions of skin translucency on the belly that are typically opaque white, suggesting a deficiency in iridophores and that it is partial hypo‐iridism of the skin (Gould and McHenry 2024; Figure 4). Skin translucency caused by an iridophore deficiency may also be visually apparent in skin sections that contain unaffected melanophores and/or xanthophores, with the skin appearing less opaque yet still coloured by the pigments of these cells if present (Nishioka 1977); the opacity may be dependent on the density of these unaffected chromatophores and their pigment content that may mask the translucency to some extent (Turner 2017).

### 5.4 Case Study: Hypo‐Iridism in Typically Green Skin

For species that are typically green (all three chromatophore types are present), a deficiency in iridophores results in the expression of a darker brown, grey, or olive skin colour, dependent on the colour mixing of the remaining chromatophores (Richards and Nace 1983). For example, *H. japonica*, typically has a bright green dorsal colour, opaque white ventrum and a golden lustre to the iris, but a type of black‐eyed mutant called the ‘Yc stock’ has dark greyish‐olive skin, semi‐translucent ventrum and dark iris, which is due to iridophores entirely lacking reflecting platelets (aniridism), while xanthophores and melanophores are unaffected (Nishioka and Ueda 1985b). In this case, the expression of olive hues is interesting as green (at least a ‘bright’ and light green hue) is primarily formed via the presence of all three chromatophore types. For this mutant, which lacks iridophores and is thus truly aniridic, the dark olive hue must arise due to the combination of yellow imparted by the xanthophores and dark brown/black/grey from the melanophores (Nishioka and Ueda 1985b). However, some authors indicate that the presence of melanophores and xanthophores together without iridophores gives rise to black/brown with a yellow tinge instead of olive green (Richards and Nace 1983), highlighting how the concentration of pigments and/or density of the chromatophores plays an important role in the final expression. In contrast, abnormal ‘olive’ variants of the black‐spotted pond frog, *Rana nigromaculata* , show olive dorsal skin which is typically green, likely due to the presence of abnormal iridophores with only a decrease in reflecting platelets number and thickness as opposed to their total absence (and thus only complete hypo‐iridism but not aniridism) (Nishioka 1977). For Schlegel's green tree frog, *Rhacophorus schlegelii* , which typically has a yellowish green dorsum, a total loss of reflecting platelets (aniridism) within the iridophores causes the skin to become a dark reddish‐brown as the skin colour is now dominated by the yellow xanthophores combined with the melanophore‐like violeophores that are dark‐violet‐black (Nishioka and Ueda 1985b). For all of these cases mentioned, the abnormal dorsal colour caused by a deficiency in iridophores is also accompanied by the blackening of the iris and increased translucency of the ventral skin. It may be difficult to identify true forms of aniridism from non‐aniridic forms of hypo‐iridism without tissue analysis, particularly of the skin, as abnormal platelets are often the cause as opposed to a true absence.

### 5.5 Hypo‐Iridsm Versus Hyper‐Melanism

The darkening of the skin caused by a deficiency in iridophores alone is difficult to distinguish from hyper‐melanism, where there is an overproduction of melanin pigment or overproliferation in melanophores, which may occur in combination with a deficiency in xanthophores and/or iridophores (Humphrey and Bagnara 1967; Jablonski et al. 2014). A key difference may be the level of darkening of the skin, with hyper‐melanic individuals being much darker than their normal type compared with individuals that have an iridophore deficiency instead (Jablonski et al. 2014).

### 5.6 Ambiguous Cases

Jablonski et al. (2014) discovered abnormally coloured green toads, *Bufotes viridis* , which typically express a pale light brown dorsum with darker brown/dark olive‐green patches, with a metallic yellow iris. They discovered juveniles which showed an abnormally darker brown dorsum with dark patches and dark irises. While the authors suggest this is axanthism, I believe this might not accurately represent the abnormality that more likely represents a deficiency in iridophores rather than xanthophores given the general darkening of the colour expression. This is noted by the typically light brown dorsal background, which must possess iridophores and melanophores, becoming darker brown in the abnormal individuals, and the metallic yellow iris, which is predominantly formed via the presence of iridophores, becoming dark brown as well. It is also noted in images provided by Jablonski et al. (2014) that the skin around the lip is typically very white, suggesting the predominance of iridophores, while being semitranslucent in abnormal individuals, further supporting this abnormality as hypo‐iridism and perhaps even aniridism more specifically. The typically green dorsal patches likely form either by the presence of all three chromatophore types or melanophores and xanthophores without iridophores, and the loss of the iridophore layer only would explain the change to a darker brown colour.

## 6 Multi‐Chromatophore Abnormalities

In some cases, an abnormal colour expression is the result of a change in multiple or all chromatophore types. These colour abnormalities are relatively more difficult to classify based on purely visual assessments. In many cases, one type of chromatophore may be primarily affected and be the most impactful on the abnormal colour expressed, while accompanied by some degree of change in the remaining chromatophores that contribute only little to the abnormality; this is a subtlety that requires tissue analysis (e.g., Nishioka and Ueda 1985a).

### 6.1 Ambiguous Case

Multi‐chromatophore deficiency is stated by Turner (2017) as the cause for an abnormally coloured adult Roth's tree frog, *Litoria rothii* . This frog's typical colouration includes a pale grey‐white dorsum (indicating the presence of iridophores and melanophores) and opaque white ventrum (indicating the presence of iridophores), yellow thighs with dark brown blotches (indicating the presence of xanthophores and melanophores), a dark brown band along the sides of the face (indicating the presence of melanophores) and irises that are metallic silver (indicating the presence of iridophores) with a rusty red region (possibly indicating the presence of xanthophores) and black venation (indicating the presence of melanophores). Importantly, this species shows an ability to adjust its dorsal surface colour temporally due to the presence of melanophores below the iridophores, changing from a ‘light phase’ (pale white) when the melanosomes are aggregated and the iridophores are exposed, to a ‘dark phase’ (dark brown) when the melanosomes are dispersed and iridophores thus covered. The colour mutant found by Turner (2017) showed relatively translucent dorsal and ventral skin (although the dorsal skin was still brown) and black irises, with no effect on thigh colouration or facial banding. Turner (2017) suggests that this is a form of hypo‐melanism, but I suggest this may not be the main deficiency, given the lack of effect on the black patches on the thighs and facial banding, which should be affected if there is a global deficiency in melanin within the skin, as well as the expression of brown dorsal skin when in its dark phase and overall capacity for colour change. Instead, the main deficiency appears to be in iridophores, resulting in the translucent ventral and dorsal surfaces, particularly in the light phase, and expression of black eyes due to the loss of silver colouration in the iris. The unaffected yellow thigh colouration would also suggest limited deficiency in xanthophores if any, except for the absence of the rusty red region of the iris. However, it remains to be determined whether this red region of the iris is formed via xanthophores or iridophores or a combination, given that iris iridophores in other animals have been shown to contain coloured pteridines typically associated with xanthophores (see Oliphant and Hudon 1993). I suggest that this individual is primarily iridophore‐deficient (complete hypo‐iridism of the skin and iris or possibly even aniridic), but potentially with a concomitant reduction in skin melanophores in some regions (partial hypo‐melanism of the skin).

### 6.2 Hypo‐Xanthism and Hypo‐Iridism With Hyper‐Melanism

Some black‐eyed mutants (Hs stock) of *H. japonica*, a species that normally has green dorsal skin, instead express a dark greyish‐brown appearance, and this is the result of a deficiency in both xanthophores (hypo‐xanthic: cells are smaller, lack pterinosomes and have smaller than usual carotenoid vesicles) and iridophores (hypo‐iridic: cells are smaller with smaller reflecting platelets), but also larger than usual melanosomes within fewer/smaller melanophores (possibly hyper‐melanic) (Nishioka and Ueda 1985b). The dark grey‐brown appearance of the dorsal skin is caused by the predominance of the colour given by the melanophores; the skin would likely express as a dark greyish‐olive if xanthophores were unaffected, as seen in the Yc stock of mutants of the same species (Nishioka and Ueda 1985b). The iridophore deficiency in particular also causes blackening of the iris and increased translucency of the ventral skin (Nishioka and Ueda 1985b). A similar abnormality has been recorded in the Mexican axolotl, *Ambystoma mexicanum* , where there is a reduction in xanthophore cells (hypo‐xanthic) and loss of iridophores (possibly aniridic) but also an increased number of melanophores (hyper‐melanic), all of which result in a dark grey to black skin colour and black irises (Frost et al. 1984; Humphrey and Bagnara 1967). These abnormal *A. mexicanum* individuals are referred to as melanoid variants or mutants rather than black‐eyed, yet it is a multi‐chromatophore abnormality much like the Hs black‐eyed mutation, which could perhaps also be referred to as a melanoid variant given the increase in size of the melanosomes.

### 6.3 Hypo‐Xanthism and Hypo‐Iridism

Adults of the Northern leopard frog, *Rana pipiens*, typically have a green dorsal surface, but black‐eyed mutants have been recorded that are dull grey in skin colour with black irises due to aniridism and axanthism, while melanophores are pigmented. It is of note that the melanophores alone create a grey skin colour as opposed to black or dark brown, which likely relates to melanin density (Ellinger 1980; Ellinger and Carlson 1978), and that blood showing through the skin in the absence of iridophores can cause the grey to show as a chocolate brown instead (Richards et al. 1969).

### 6.4 General Pigment Reduction

Other abnormalities may involve deficiency in the pigment content of chromatophores but not a deficiency in cell number. For example, a pale mutation found in the oriental fire‐bellied toad, *Bombina orientalis*, was due to normal chromatophore presence but with reduced organelles or a partial defect in pigment biosynthesis (Ellinger 1980; Ellinger and Carlson 1978). This caused typically brown dorsal regions to become light grey, and typically dark green regions to become light green, suggesting complete hypo‐melanism, hypo‐iridism and hypo‐xanthism of the skin but not true amelanism, aniridism and axanthism.

## 7 Colour Change

Determining whether wild frogs possess a chromatophore abnormality can be challenging given that some species may show natural short‐ or long‐term colour changes that are triggered by environmental conditions and result in temporary changes in chromatophores in the skin (Bagnara and Hadley 1969; Bagnara et al. 1968; Nielsen 1978; Taylor and Bagnara 1972) but also probably the iris (Oliphant et al. 1992). For example, adult *L. aurea* males show a prolonged yet subtle yellowing of skin during the breeding season, which is likely triggered by hormones leading to increased xanthophore pigment synthesis (i.e., morphological colour change), while the stony‐creek frog, *Litoria wilcoxii* , shows rapid skin yellowing caused by the temporary aggregation of melanosomes within melanophores (i.e., physiological colour change) that reveals the xanthophore pigment (see Gould and McHenry 2024). Similarly, an increase in iridophore number and greater regularity of reflecting platelet orientation has been recorded in the reed frog, *Hyperolius viridiflavus nitidulus* , causing skin to change from brown (due to the presence of melanophores) to a brilliant white (as the melanophores effectively become covered and interact little with incoming light) during the dry season (Kobelt and Linsenmair 1986). Individuals with abnormal colouration may look similar to unaffected frogs of the same species but only under certain environmental conditions if temporary colour change is possible and thus only distinguished by (i) examining the individual under all conditions (i.e., environmental triggers and seasons) and (ii) comparing both skin and eye colours with normal individuals of the same species to detect regions that do not have the capacity for colour change and may thus be more reliable visual identifiers of an abnormality. Additionally, desiccation, disease, and dietary deficiencies can also result in temporary changes in tissue colouration, which must be considered.

### 7.1 Case Study: Colour Change With Colour Abnormalities

**FIGURE 5:**
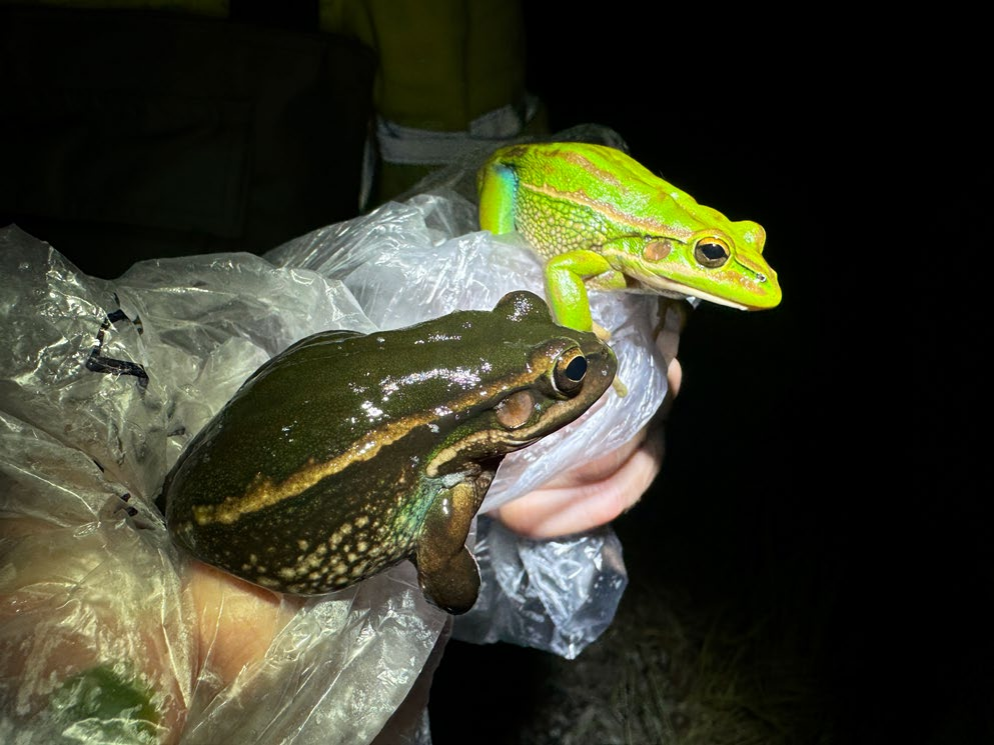
Natural colour change in green and golden bell frogs, *Litoria aurea* , caused by temperature/lighting. The dorsal skin of adults darkens from a light pea green to a dark olive green as melanosomes in the melanophores disperse and cover the overlying iridophores.

A clear example is *H. japonica* , which typically possesses a green dorsal surface that naturally becomes grey/brown dependent on environmental conditions such as lighting and thus shows rapid colour change (Nishioka and Ueda 1985b). This is the result of melanosomes in the melanophores dispersing from the body of the cell into arms that extend upwards into the dermal layer around the sides of the iridophore and subsequently finger‐like dendrites that terminate over the iridophores, masking the light reflecting effect of the iridophore, darkening the skin and interrupting the effect of the iridophore on the xanthophore pigment above it (Bagnara et al. 1968; Taylor and Bagnara 1972). The Yc stock of black‐eyed mutants of this species, which are aniridic, do not undergo rapid colour change but remain dark grey‐olive (Nishioka and Ueda 1985b), alluding to the role of yellow xanthophores in combination with melanophores in creating the dark green hue of these abnormal mutants without any contribution from iridophores. This connection between dark greens and xanthophore/melanophore combinations is also supported by the natural colour change observed in *L. aurea* , which changes from pea green to dark green as the iridophores become masked by melanin under dark/cold conditions (Figure 5). This loss of colour change may be a useful diagnosis to determine the type of chromatophore abnormality present as it suggests a deficiency in iridophores; the movement of the melanosomes in the melanophores that occurs with colour change would otherwise have an impact and change the skin colour unless the melanophores were also abnormal. In contrast, blue mutant variants of *H. japonica*, which are hypo‐xanthic/axanthic, show blue skin under light conditions that rapidly changes to brown/grey under dark conditions; this is much like the colour of typical individuals under dark conditions and indicates that the blue colour produced by the iridophore is temporarily stopped by melanosomes covering the iridophores (Nishioka and Ueda 1985a). A caveat to this is that, in at least some species, the melanosomes within the melanophores migrate above the overlying xanthophores as opposed to just the iridophores or when iridophores are absent (e.g., Kindermann and Hero 2016); this is of note as much of the literature indicates the melanosomes migrate above the iridophore only (Bagnara et al. 1968; Taylor and Bagnara 1972).

## 8 Documenting Colour Abnormalities

The visible appearance of abnormally coloured amphibians must be described in detail to assist in the identification of the likely chromatophores that are affected, particularly if histological analyses of tissues cannot be performed. This includes an assessment of (1) the tissues involved, including skin (dorsum, ventrum, banding patterns, thigh flash colouration, legs and face) and eyes (iris and retina), (2) the typical colour range of each tissue section of the target species and (3) the capacity for natural/temporary colour change for each tissue over time (e.g., light vs. dark conditions) or the effect of dietary intake of pigments (most notably the impact of carotenoid intake on the colour of xanthophores/erythrophores). This should be used to then form the basis of a visual diagnosis of the abnormality, including whether it may involve (i) hypo‐melansim, hypo‐xanthism or hypo‐iridism, and (ii) whether it is partial or complete deficiency for each tissue type.

Abnormal variants have been presented in the literature as stand‐alone images without comparative images of individuals from the same sex, life stage and population also provided. In the absence of these visual comparisons, it may be difficult for readers to interpret the extent of colour abnormality that is being expressed by individuals. When possible, individuals with abnormal colouration should be photographed with typical individuals, thus allowing for direct comparisons to be made. This is apparent in the work conducted by Brito‐Zapata (2021) and Jablonski et al. (2014), which visually depict both typical and abnormal individuals for all pigmented tissues, including ventral, side and dorsal views. If possible, the animals should be photographed side by side, thereby controlling for the potential effect of lighting (direction, colour temperature and intensity) and animal positioning on colour quality and appearance. The individuals should be of the same sex, size and life‐history stage as individuals with colour aberrations, in order to remove natural variability in skin colouration caused by these biological attributes. If species show colour morphism, it may be necessary to show comparisons of each typical colour morph with the abnormal variant. Additionally, the work of Turner (2017) clearly shows the importance of photographing the potential change in colouration of species with this capacity. Each of these steps should be standard procedure when reporting on colour abnormalities in amphibians.

## 9 Conclusion

Identifying the chromatophores involved in colour abnormalities is challenging, particularly if it is solely based on a visual comparison of atypical versus typical colourations for a given species. This is because colour expression among amphibians is often the result of light interacting with multiple chromatophore types, whereby the same chromatophore deficiency can have entirely different outcomes between tissue regions and species dependent on the presence and combination of other chromatophore types that are unaffected. Given that most records of colour aberrations will continue to be based on visual assessments without the procurement of tissue samples as they are generally opportunistic, standard terminology and detailed descriptions are needed to deduce the potential chromatophore deficiencies involved. The detailed exploration of chromatophore deficiencies in this review will assist researchers in assessing abnormally coloured amphibians.

## Author Contributions

**John Gould:** conceptualization (lead), investigation (lead), project administration (lead), writing – original draft (lead), writing – review and editing (lead).

## Conflicts of Interest

The author declares no conflicts of interest.

## Data Availability

There is no data associated with this paper.
